# A Novel Master–Slave Interventional Surgery Robot with Force Feedback and Collaborative Operation

**DOI:** 10.3390/s23073584

**Published:** 2023-03-29

**Authors:** Yu Song, Liutao Li, Yu Tian, Zhiwei Li, Xuanchun Yin

**Affiliations:** 1 Tianjin Key Laboratory for Control Theory and Applications in Complicated Industry Systems, School of Electrical Engineering and Automation, Tianjin University of Technology, Tianjin 300384, China; 2 School of Engineering, South China Agricultural University, Guangzhou 510642, China

**Keywords:** haptic interface, vascular intervention robot, magnetorheological fluid (MRF)

## Abstract

In recent years, master–slave vascular robots have been developed to address the problem of radiation exposure during vascular interventions for surgeons. However, the single visual feedback reduces surgeon immersion and transparency of the system. In this work, we have developed a haptic interface based on the magnetorheological fluid (MRF) on the master side. The haptic interface can provide passive feedback force with high force fidelity and low inertia. Additionally, the manipulation of the master device does not change the operating posture of traditional surgery, which allows the surgeon to better adapt to the robotic system. For the slave robot, the catheter and guidewire can be navigated simultaneously which allows the two degrees of action on the catheter and axial action of a guidewire. The resistance force of the catheter navigation is measured and reflected to the user through the master haptic interface. To verify the proposed master–slave robotic system, the evaluation experiments are carried out in vitro, and the effectiveness of the system was demonstrated experimentally.

## 1. Introduction

The improvements in healthcare services result in a longer life expectancy and a higher number of aged individuals, who are inevitably affected by cardiovascular diseases [1]. The treatment of cardiovascular diseases has gone from open surgery techniques to minimally invasive intervention (MISI) with technological development [2]. In MISI, the surgeon passes the catheter/guidewire used for treatment through the aorta of the lower extremity, moves the catheter to the location of the lesion, and places a stent or injects a drug. During the procedure, the surgeon feels the resistance of the catheter in the blood vessel through haptic feedback and observes the position of the catheter through medical fluoroscopy. Medical fluoroscopy provides visual feedback while compromising the health of the interventionalists and increasing the risk of cancer, cataracts, and other diseases. Even when interventionalists wear radiation-protective lead suits, their hands and face are still exposed to radiation, and wearing heavy lead suits for a long time will cause musculoskeletal strain.

Interventionalists’ dilemma motivates the development of master–slave vascular interventional robots. The advantage of the master–slave structure is that it separates the surgeon’s operating scenario, allowing the surgeon to complete the MISI without radiation. Ref. [3] notes that robotic-assisted vascular interventions reduce 97.1% of the radiation exposure of surgeons compared to conventional procedures. Some commercial robotic catheter operating systems, all employing the master–slave control architecture, have demonstrated safety and efficacy in endovascular surgery, such as Corpath 200 and Corpath GRX [4], in which the surgeon operates the catheter/guidewire located on the patient’s side via a handle and touch screen inside the radiation-shielded compartment.

Force feedback allows the surgeon to move away from the single visual dependence and reduce resistance during delivery in the procedure, which is significant for vascular interventional robots [5]. Force feedback has also been developed by some research groups. In ref. [6], using sensors and motors to construct haptic feedback, the experiments demonstrated that the robotic system applied lower force values during operation compared to manual, with an average reduction of 76%. Refs. [7,8,9] used a motor to provide repulsive force and realize force feedback. However, due to backlash and resolution issues problems, the motor-based haptic feedback system has weakness in describing the viscosity of blood or viscoelastic properties of organs.

In addition to using motors, some research groups use new intelligent materials to generate haptic feedback. The shape memory effect and linear braking properties of shape memory alloys (SMA) are suitable for haptic feedback. Ref. [10] uses SMA which could offer haptic feedback in a novel handheld instrument. The rheological properties of electrorheological fluid (ERF) change dramatically and reversibly when subjected to the action of an external electric field. The multi-DOF master handle was developed using ERF, which can give haptic feedback to the surgeon in surgery [11,12]. Although the material properties of ERF are suitable for the development of haptic feedback devices, the activation of ERF requires high voltage [13], and it needs strong insulating materials, which also create obstacles to the safety of the equipment. Like the ERF, the rheological properties of the magnetorheological fluids (MRF) change reversibly in response to changes in the external magnetic field. A fully hand-immersive haptic interface is proposed using MRF, and the effectiveness of the proposed device is verified by psychophysical experiments [14]. A haptic feedback device using a magnetic powder clutch is proposed by Yu [15]. Ref. [16] proposed a haptic feedback device based on MR-break and a pneumatic actuator. A medical application haptic interface based on MRF-clutch is proposed by Najmaei [17,18]. Our group has developed a haptic feedback device for vascular intervention surgery [19,20]. It is important to note that the device we developed not only provides haptic feedback but also does not change the surgeon’s traditional surgical posture. Considering that vascular catheter insertion surgery has a steep learning curve and is heavily dependent on operator experience [21], the design of our group will facilitate surgeons’ adaptation to the robotic operating system and improve the success rate of the procedure. However, the size of the device is too large for installation and produces little force feedback [22], which limits the application of the device.

In this paper, we have developed a new generation of haptic feedback interfaces. Compared to the previous generation of haptic interfaces, we use a disc immersed in MRF to obtain sufficient resistance to achieve haptic feedback, simplifying the design structure and reducing the size of the device. We use a rigid catheter in the same position as the conventional procedure, reducing the surgeon’s adaptation time. We have simulated and analyzed the magnetic field to verify the validity of our design. Considering that the fluid properties of MRF vary with the magnetic field, we use the Bingham model to describe the fluid and analyze our design. Finally, we set up two experiments to validate our developed device. In the first experiment, we used different speeds to test the relationship between speed and resistance at low-speed levels. In the second experiment, we used in vitro model experiments to verify the effectiveness of the developed equipment.

## 2. Materials and Methods

### Magnetorheological Fluid 

**Figure 1:**
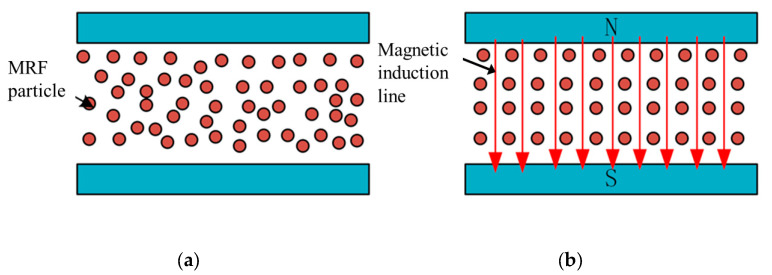
(**a**) Material properties of MRF without magnetic field. (**b**) Material properties of MRF with a magnetic field.

Magnetorheological fluids comprise three main components: magnetic particles, carrier fluid, and additives. The preparation of magnetorheological fluids includes two components: the solid phase and the liquid phase. We use the MRF-112EG made by Lord Company in this work. Due to its unique material composition, the material properties of MRF vary with the external magnetic field. MRF can reversibly change from a Newtonian fluid to a non-Newtonian fluid in a millisecond. Figure 1 shows the material properties of MRF.

**Figure 2:**
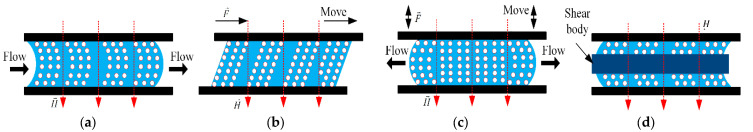
MRF working mode. (**a**) Flow mode; (**b**) Shear mode; (**c**) Squeeze mode; (**d**) Bi-shear mode.

When influenced by the magnetic field, the particles in MRF will be aligned along the magnetic induction lines, the attraction between the particles becomes larger, and the viscosity of the material is changed. MRF usually works in four modes: flow mode, shear mode, squeeze mode, and bi-shear mode. Figure 2a shows the operating state of the flow mode, in which the container is relatively stationary and the MRF flows with the velocity of the flow being influenced by the external magnetic field. Figure 2b shows the operating state in shear mode, in which the two sides of the container are displaced relative to each other, and the chain structure of the fluid is changed due to the shear force. Figure 2c shows the squeeze mode, where the upper and lower walls are in relative motion along the magnetic field direction. As the container space changes, the fluid is squeezed to flow out both sides. Figure 2d shows the bi-shear mode, where the object passes through the MRF and the top and bottom ends of the object are in shear mode.

The shear effect of magnetorheological fluid has stable characteristics in the low-speed state. The research of ref. [23] shows that the viscosity characteristics of MRF will change in the long-term high-speed shear working state, and the MRF viscosity will increase. The main working state of the MRF in the equipment we developed is mainly a low-speed shear state, and the continuous working time is not long, so we think the state of MRF is stable.

## 3. Master–Slave Robot System

### 3.1. Master Robot System

In conventional interventional procedures, the surgeon mainly uses his fingers to move the catheter/guidewire axially and radially to navigate it in the vessel. During procedures, the surgeon uses visual feedback (medical fluoroscopy) and haptic feedback (resistance to the catheter) to determine the state of the catheter/guidewire within the vascular. We have developed a vascular interventional robot in which the surgeon manipulates a rigid catheter without changing the traditional manipulation habits, and more significantly, it can restore the resistance of the catheter in the vessel. In addition, the developed equipment can capture the surgeon’s manipulation movements of the rigid catheter and send motion signals to the slave manipulator.

#### 3.1.1. Design of Haptic Feedback Interface

**Figure 3:**
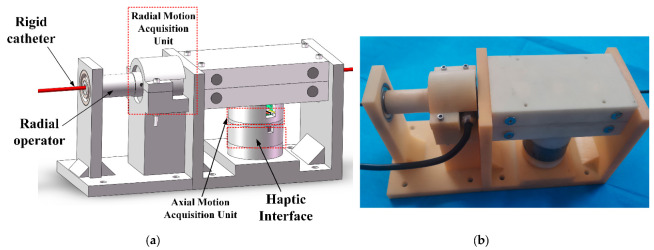
(**a**) Master manipulator CAD drawing. (**b**) Master manipulator physical drawing.

**Figure 4:**
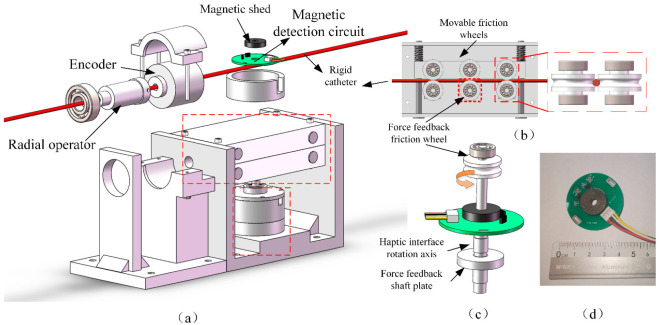
Exploded view of developed master manipulator. (**a**) Master manipulator. (**b**) Structure of axial delivery unit. (**c**) Haptic feedback interface. (**d**) Hall Encoder.

In this work, we have developed a haptic feedback interface for vascular intervention robots by MRF. Figure 3 shows the CAD drawing and physical drawing. Figure 4 shows the exploded view of the developed manipulator.

The master manipulator uses two encoders to acquire the surgeon’s manipulation signal at the catheter and send it to the slave. When the surgeon delivers the rigid catheter axially, the catheter rotates the Hall encoder through the friction wheel to achieve the measurement of the axial movement. The surgeon manipulates the rotation of the slave catheter using a rotating tube, which is connected to an encoder to detect the radial rotation movement. The removable friction wheel design allows for easy loading and unloading of the device, and the force feedback friction wheel is connected to a haptic interface for replicating the resistance of the slave catheter.

#### 3.1.2. Principle of Haptic Interface

In conventional vascular interventions, the surgeon manually manipulates the catheter/guidewire, feels the resistance to the catheter in the vessel, adjusts the posture of the catheter, and smoothly inserts it. The resistance at the catheter during the procedure is an important reference for the safe performance of the procedure. Excessive resistance can cause the catheter to puncture a blood vessel and pose a risk to the procedure. So, we developed a robot that can give feedback on the resistance at the catheter and give the surgeon a reference.

We use a magnetorheological fluid (MRF) to generate resistance haptic feedback, and the viscosity of this material is influenced by the magnetic field. When MRF is in a magnetic field-free environment, the fluid viscosity is low and the mechanical properties are in accordance with Newtonian fluid properties, which can be expressed as:

(1) $$\tau =\eta \cdot \dot{\gamma }$$

where τ is the shear force of the MRF, η is the viscosity coefficient of the MRF, and $\dot{\gamma }$ is the shear rate. When the fluid is subjected to a magnetic field, the fluid viscosity increases, and the mechanical properties conform to the Bingham fluid properties, which can be expressed as:

(2) $$\tau_{H}=\tau_{0}\left(H\right)+\eta \cdot \dot{\gamma }$$

where $\tau_{0}\left(H\right)$ is a constant related to the strength of the applied magnetic field.

**Figure 5:**
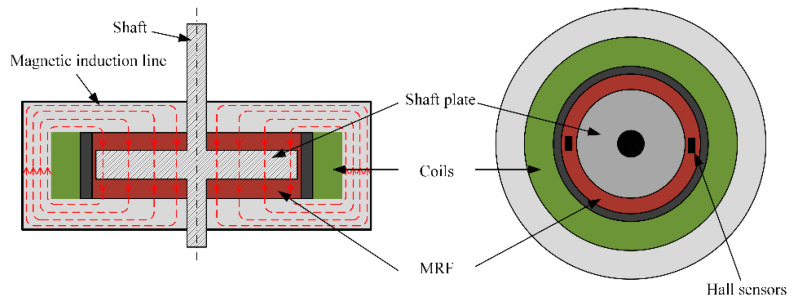
Schematic diagram of disc and magnetic susceptibility lines.

**Figure 6:**
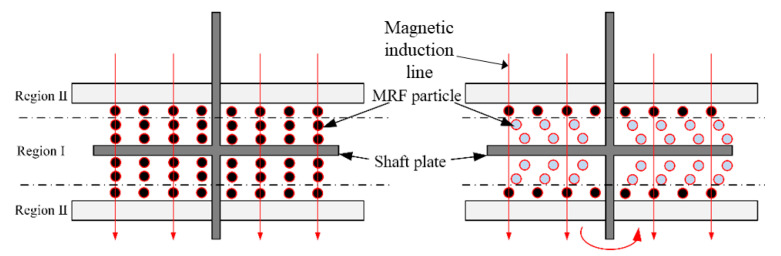
Particle distribution when the disc is rotating.

Figure 5 shows the haptic interface we developed which immerses a disc in the MRF, and the disc is oriented perpendicular to the applied magnetic field. When no current is applied, the viscosity of MRF is smaller and the viscous resistance to the rotation of the disc is smaller. When an electric current is applied, the MRF is converted to Bingham fluid by the magnetic field, the viscosity increases, and the chain-like structure between the particles hinders the rotation of the disc, forming a resistance. Figure 6 shows the profile of the particle shear. The operating mode of MRF in the haptic interface is the double shear mode, which provides sufficient damping force.

**Figure 7:**
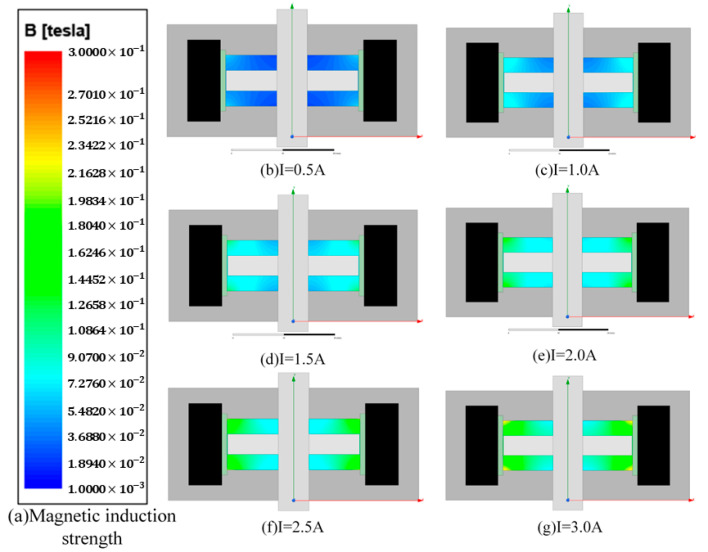
ANSYS MAXWELL simulation results.

To determine the effect of the magnetic field on the MRF, we analyzed the magnetic field using ANSYS maxwell software. Firstly, we set the current in the simulation coil to 0.5 A, 1.0 A, 1.5 A, 2.0 A, 2.5 A, and 3 A, and we set the material to Q235 steel and the rotating shaft to non-permeable stainless steel. Figure 7 shows the simulation results; in the same horizontal direction, the closer to the coil, the greater the intensity, the axial variation is obvious, and the magnetic field strength can meet the need to activate the MRF fluid.

**Figure 8:**
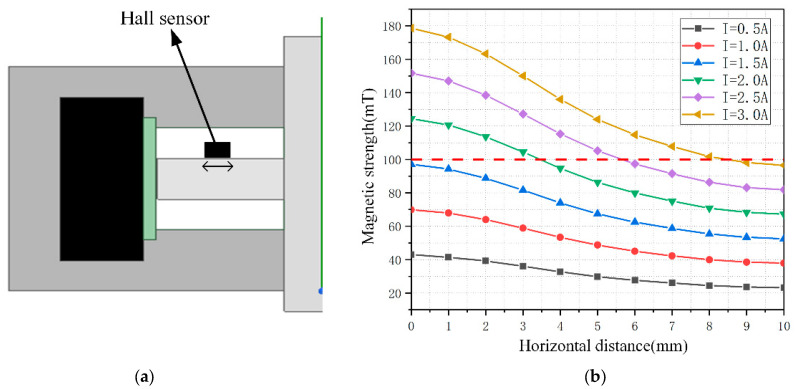
(**a**) Local profile of haptic interface simulation. (**b**) Relationship between field strength and horizontal displacement of recording points.

A single area in the upper left corner of the MRF was selected for analysis, which has a horizontal distance of 10 mm and a height of 3 mm. To detect a larger magnetic field range, the hall sensor is mounted close to the disc position, as shown in Figure 8. Considering that the thickness of the haptic interface along the magnetic field direction is 1.6 mm and the detection point is located at the center of the sensor, the magnetic induction intensity in the horizontal direction at 0.8 mm from the disc is calculated and recorded. The Hall sensor we used is Honeywell’s SS49E linear Hall effect sensor, and the sensor size is 4.1 mm × 3.0 mm × 1.6 mm. The relationship between the horizontal translation distance and the magnetic induction intensity was recorded for current values in steps of 0.5 A in the range of 0.5 A to 3.0 A, and the results were obtained as shown in Figure 8. Considering the width of the Hall sensor is 3.0 mm, the sensor can be moved in the horizontal direction in the range of 1.5–8.5 mm, and the final selection of the installation position is the closest to the rotation axis, which can detect the magnetic field generated by a 0–3.0 A current.

#### 3.1.3. Haptic Interface Force Sensory Model Analysis

To model the feedback force of the haptic interface and thus derive the relationship between the output torque and the strength of the magnetic field applied by the coil, the following assumptions are made for the haptic interface before calculation.

(1) Considering that MRF particles produce attractive forces along the magnetic field direction, we assume that the excitation magnetic field generated by energizing the electromagnetic coil is a uniform magnetic field.
(2) The effect of gravity on the motion of the MRF is neglected, and the effect of gravity on the circumferential rotation of the axial disc is neglected.
(3) Assuming that the distance between the side of the disc and the coil support is small enough, the magnetorheological fluid is distributed above and below the disc.

**Figure 9:**
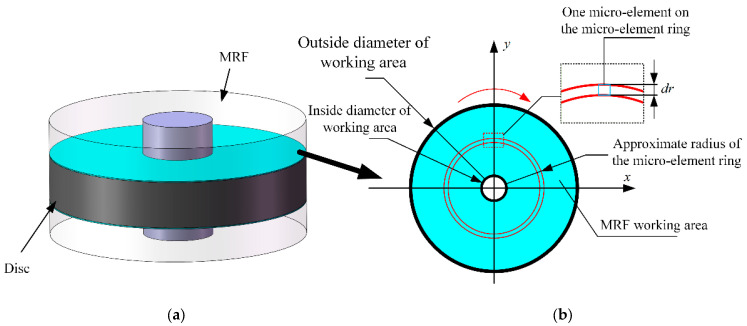
(**a**) Magnetorheological fluid area. (**b**) Upper view of the MRF region generating resistance.

Figure 9a represents the abstracted MRF region, where the green portion is the MRF with the resistance exerted on the disc, and the output resistance of the disc is provided by this portion. Figure 9b shows the upper view of the fluid particles in this part. After applying the magnetic field, the working area formed by the force on the MRF particles is circular in shape.

**Figure 10:**
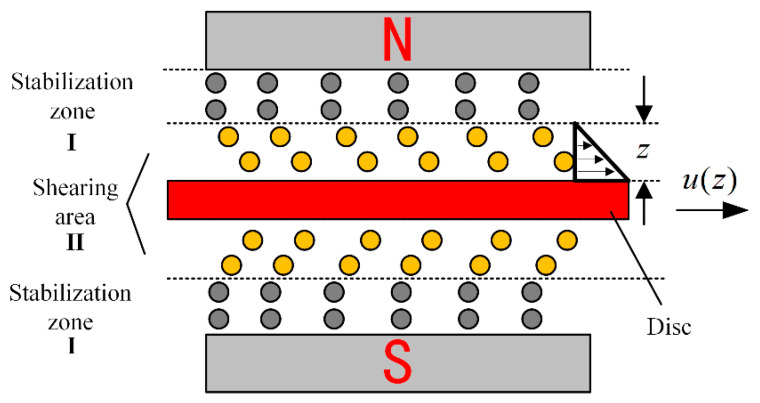
MRF shear force versus velocity distribution.

The inner and outer longitudes of the ring are $R_{1}$ and $R_{2}$, respectively. The working region of the MRF is abstracted as a micro-ring of width dr. When dr is assumed to be small enough, its radius is approximated as r. Then, the torque of the shear force of this micro-ring is:

(3) $$dT_{\tau }=r\cdot dF_{\tau }\;$$

where $dF_{\tau }$ is the total shear stress generated by the microcircle. Assuming that the shear stresses in each microcircle are equal for each microelement of the microcircle ring, the shear stresses in each microelement are:

(4) $$\tau =\tau_{dy}\left(H\right)+\eta \cdot \dot{\gamma }$$

where $\tau_{dy}\left(H\right)$ is the dynamic yield stress, η is the viscosity factor, and $\dot{\gamma }$ is the shear rate along the magnetic field direction (z-direction). The relationship between shear stress and velocity distribution of the MRF is shown in Figure 10, which yields:

(5) $$\dot{\gamma }=\frac{du\left(z\right)}{dz}$$

The total shear stress of the micro-circular ring is:

(6) $$\begin{array}{ll} dF_{\tau } & =\tau \cdot dS\\ & =\tau \cdot \left(2\pi r\cdot dr-\pi {\left(dr\right)}^{2}\right)\\ & \approx 2\pi r\tau \cdot dr \end{array}$$

Substituting Equation (6) into Equation (3) yield:

(7) $$\begin{array}{ll} dT_{\tau } & =r\cdot dF_{\tau }\\ & =2\pi r^{2}\cdot \left(\tau_{dy}\left(H\right)+\eta \cdot \frac{du\left(z\right)}{dz}\right)dr \end{array}$$

The working area of the MRF consists of numerous micro-rings with radii from $R_{1}$ to $R_{2}$, so the calculation of the shear stress output torque for the whole shaft disk requires the integration of dr from $R_{1}\to R_{2}$:

(8) $$\begin{array}{c} T_{\tau }=\int_{R_{1}}^{R_{2}}2\pi r^{2}\cdot \left(\tau_{dy}\left(H\right)+\eta \cdot \frac{du\left(z\right)}{dz}\right)dr\\ =\frac{2}{3}\pi \left(\tau_{dy}\left(H\right)+\eta \cdot \frac{du\left(z\right)}{dz}\right)\left(R_{2}^{3}-R_{1}^{3}\right) \end{array}$$

The relationship obtained above is the torque versus magnetic field strength for an operating region. The operating mode of the haptic interface of this design is a double shear mode, which produces a rheological effect in both the upper and lower parts of the disc, so that the actual output torque of the shear force of the disc is twice that of a single working area. Additionally, the resistance torque output from the haptic interface is the sum of the frictional torque $T_{s}$ generated by the frictional resistance of the mechanical structure and the torque generated by the shear force of the magnetorheological fluid as:

(9) $$T=\frac{4}{3}\pi \left(\tau_{dy}\left(H\right)+\eta \cdot \frac{du\left(z\right)}{dz}\right)\left(R_{2}^{3}-R_{1}^{3}\right)+T_{s}$$

### 3.2. Slave Robot System

The slave of the vascular intervention robot was developed to receive the action signal from the master manipulator and to complete the catheter/guidewire motion. In addition, the slave manipulator should detect the resistance condition of the catheter in the vessel and send a reference signal to the master for force feedback. To achieve the coordinate of the catheter/guidewire, the device should also have the function of clamping and relaxing movement, simulating the gripping action of the surgeon.

#### 3.2.1. Structure and Principle of Slave Manipulator

**Figure 11:**
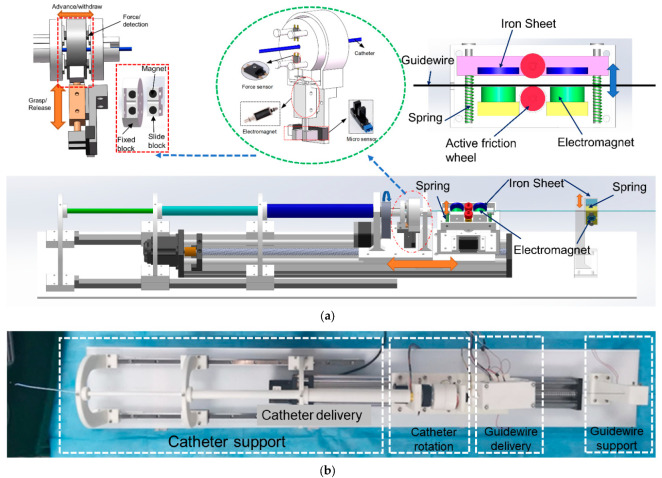
(**a**) Slave manipulator CAD structure. (**b**) The developed equipment.

The slave robot realizes axial and radial movements on the catheter and sets only axial movements on the guidewire. Figure 11 shows the slave manipulator we developed. To prevent the catheter/guidewire from bending during delivery, we set up a sleeve structure at the front end of the equipment to prevent the catheter from falling and guidewire support at the back end of the equipment. The guidewire support also has the function of clamping and releasing the guidewire, which facilitates the cooperative movement of the catheter/guidewire. In the guidewire delivery unit, the movable slide rod is installed with two iron pieces and a driven friction wheel; when attracted by the electromagnet which is fixed on the other side, the iron piece will drive the slide rod to move, and the driven friction wheel and the active friction wheel become close together, clamping the guidewire. The stepping motor drives the friction wheel to rotate, driving the guidewire forward and backward at the clamp state. When the electromagnet is de-energized, the spring ensures that the sliding rods are separated from each other. In the separated state, the guidewire support unit is responsible for clamping the guidewire to ensure that it does not move. Like the clamping principle of the catheter delivery unit, the clamped and released movement of the guidewire support unit is realized by a solenoid and an iron piece.

In addition to replicating the catheter resistance, the slave device also needs to transmit the resistance of the intravascular catheter to the master equipment, which serves as a force feedback reference. We implement the force measurement in the catheter rotation unit. In the process of catheter delivery, the catheter rotation unit clamps the catheter, and the stepping motor drives the entire slide to move with a screw drive to achieve the forward and backward delivery of the catheter. The clamping and loosening of the catheter are achieved by the push–pull solenoid and design structure. The state of relaxation and clamping is detected by the photoelectric sensors. Two permanent magnets of opposite polarity are mounted separately in the fixture to supplement the clamping force to ensure stable clamping. The gripper is mounted next to four force sensors in the structure and has a certain degree of freedom of offset. When the catheter is under resistance, the gripper is offset and touches the force sensors, and the resistance is detected.

#### 3.2.2. Cooperative Operation of Slave Manipulator

**Figure 12:**
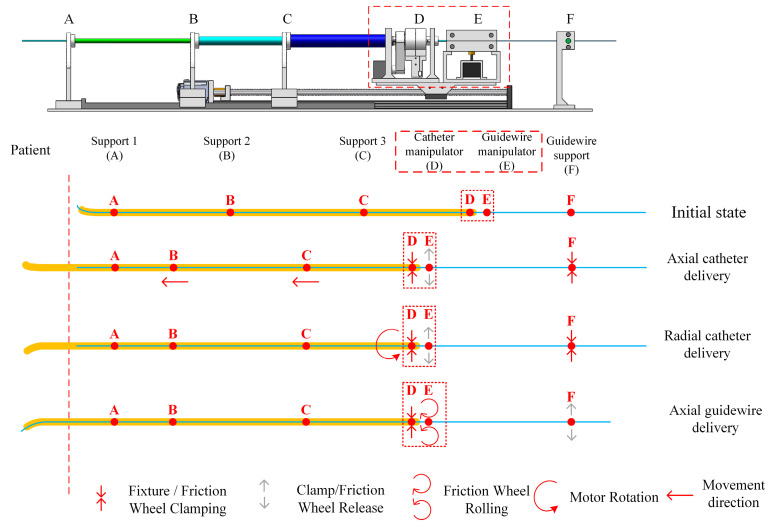
Catheter/guidewire cooperative operation.

Figure 12 illustrates the catheter/guidewire delivery process. The catheter/guidewire delivery coordination of the vascular interventional robot from the surgical robot is achieved by the interplay of the catheter manipulation unit, the guidewire manipulation unit, and the stepper motors and clamps in the guidewire support unit.

The starting points of the three sections of the catheter support units are marked as points A, B, and C, respectively; point D is the clamping point of the fixture of the catheter manipulation unit, E is the clamping point of the friction wheel of the guidewire manipulation unit, F is the clamping point of the fixture of the guidewire support unit, and parts E and F are fixed with the slide table for common displacement. When the catheter is delivered axially forward from the end surgery robot, the clamp of the catheter manipulation unit clamps the catheter, and the slide table drives the whole catheter and the guidewire manipulation unit to be displaced, and the catheter is delivered forward, at which time the clamp of the guidewire support unit clamps to ensure that the guidewire is not displaced. When the catheter is delivered radially, the clamps in the catheter manipulation unit clamp the catheter and the stepper motor drives the clamps to rotate to achieve catheter rotation, at which time, the clamps in the guidewire support unit clamp the guidewire. When axially delivering the guidewire, the clamps of the guidewire support unit are released, and the friction wheel of the guidewire manipulation unit grips the guidewire, which is driven by the stepper motor to rotate the friction wheel to realize the guidewire delivery, and the clamps of the guide manipulation unit are closed during this process.

### 3.3. Force Feedback Strategy

In our system, the resistance of the slave catheter is detected at the slave device and transmitted to the master device, which receives the resistance signal and through a different currently activated MRF generates a different resistance to the surgeon as a reference.

In the slave device, the friction force to overcome the catheter sheath is about 0.8 N, and the maximum contact force that the human vasculature can withstand is 0.12 N [24]. Therefore, we set a force interval of 0.8–0.92 N as our feedback interval, and when the slave detects a force within this range, the slave device will transmit the signal to the master device to generate force feedback.

When the force value of 0.8–0.86 N is detected by the sensor at the slave, the force includes the viscous resistance of blood and the tiny friction between the catheter and blood vessel, and there is no risk of piercing the blood vessel and no current through to coil of the master manipulator. When 0.86–0.89 N is detected, it means that there is minor friction between the catheter and the blood vessel wall, and the risk of piercing the blood vessel is small. A current is applied to the master device to produce a magnetic field of 20 mT, generating a feedback force of about 1.8 N. When 0.89–0.92 N is detected, it means that the catheter tip and the blood vessel wall occur extrusion, and the risk of puncturing the vessel wall is greater. A current is applied to the master device to produce a magnetic field of 50 mT, generating a feedback force of about 2.5 N. When the detected force is greater than 0.92 N, this will have exceeded the safety force threshold that the vessel can withstand. A current is applied to the master device to produce a magnetic field of 80 mT, generating a feedback force of about 3.5 N. When a resistance of more than 0.95 N is detected on the slave manipulator, a current is passed to the master end to generate a magnetic field of 100 mT and the slave manipulator is inactive, which is considered a dangerous operation. According to the research related to human touch, the smallest change force that can be perceived by the human hand is 0.35 N [25], so our force feedback interval is reasonably designed.

We use two STM 32 microcontrollers for master and slave control, and the master and slave communicate via the RS485 bus.

## 4. Experimental and Results

### 4.1. Haptic Feedback Calibration Experimental

Due to the influence of friction and sliding factors, the performance of the master manipulator cannot be derived from theoretical calculations alone, and we use experimental methods to analyze its resistance characteristics.

**Figure 13:**
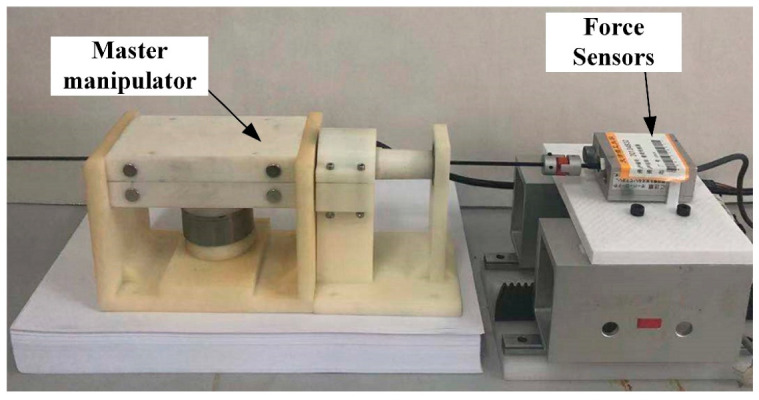
Experimental setup.

**Figure 14:**
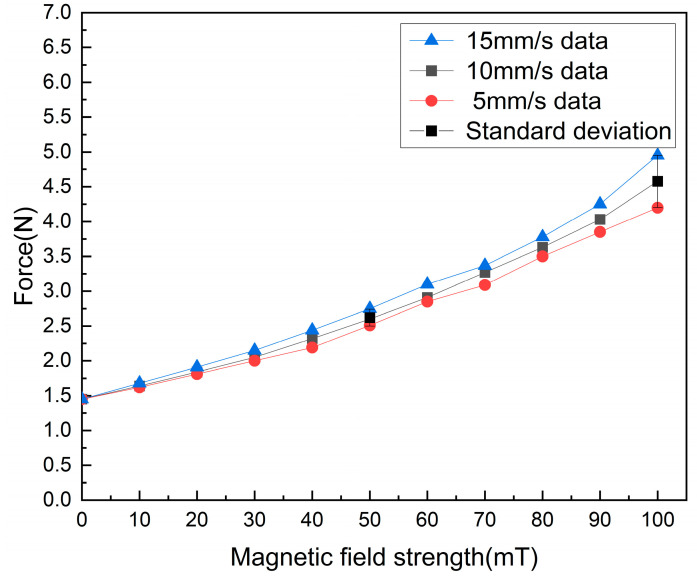
Validation experimental data from the master manipulator.

The experimental setup is shown in Figure 13, where the developed manipulator is placed on a part, a load cell and a rigid catheter are at the same height and capable of measuring the delivery resistance, the load cell being driven by a screw slide with a stepper motor. In the experiment, the axial delivery speed of the catheter was set to 10 mm/s, and the magnetic field strength was changed by changing the current magnitude, and the force information output from the force sensor was recorded. The data acquisition card allows the acquisition of the load cell data. Figure 14 shows the experimental results. We fit the 10 mm/s data yields:

(10) $$F=0.0002B^{2}+0.0149B+1.46$$

where F represents the haptic force, and B represents the magnetic induction strength. As seen in Figure 14, our device can generate a feedback force of about 1.5 N–4.65 N. In conventional surgery, the surgeon holds the end of the catheter in his hand and the resistance to the delivery of the catheter is synthesized by three types of forces: (1) The viscous force between the blood and the catheter. (2) The friction force between the catheter and catheter sheath. (3) The impact force between the vessel wall and the tips of the catheter. Under dynamic and static conditions, the bonding force usually ranges from 0 to 2 N, with a few reaching close to 4 N [26]. Ref. [27] uses commercial force feedback equipment, and it can produce 3.3 N of force feedback. Therefore, we believe that our force feedback interval is reasonable.

In the validation of the master haptic device, we verified the force feedback performance generated by the device at different delivery speeds, and we found that there was some difference in the resistance of the device at different speeds under the same magnetic field. We choose 10 mm/s to fit the data, which is the same as our delivery speed from the slave side.

In our force feedback strategy, we choose to pass a current to the master end to generate a magnetic field of corresponding strength when the slave end exceeds a set threshold, and the resistance generated by the MRF changes with the strength of the magnetic field, so that the surgeon feels the resistance feedback and thus determines the state of the catheter in the vascular.

We found that the stronger the magnetic field, the greater the difference produced between different speeds, so we choose different magnetic field strengths of 20 mT, 50 mT, and 80 mT, which produce a resistance of about 1.8 N, 2.5 N, and 3.5 N, with more stable feedback force performance at low speeds.

### 4.2. Slave Manipulator Performance Evaluation

**Figure 15:**
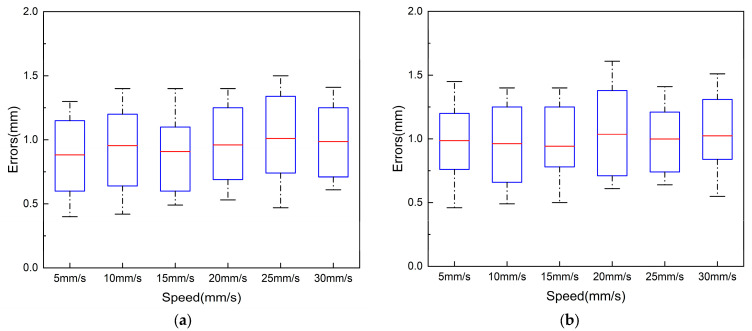
(**a**) Axial catheter delivery error. (**b**) Axial guidewire delivery error.

To ensure the safe performance of robotic vascular interventions, the slave manipulator is required to be able to deliver the catheter/guidewire precisely in response to the action commands of the master operator. In this paper, we verified the error of delivering 1000 mm at different delivery speeds. In the experiment, we verified the error of 5 mm/s, 10 mm/s, 15 mm/s, 20 mm/s, 25 mm/s, and 30 mm/s. We repeated the experiment 10 times at different speeds, and the error data of these ten times are shown in Figure 15.

In our catheter displacement experiments, due to the tip of the catheter being curved, in order to ensure the accurate measurement of axial displacement for detection, detection marker points were placed at the tip of the straight part of the catheter. Experimentally delivered commands are output via PWM signals from the STM32 at the controller to control the rotation of the stepper motor responsible for catheter and guidewire delivery. The delivery distance between the catheter and the guidewire was set to 1000 mm. During the experiment, different delivery speeds were set, and the delivery error was recorded at the end of each trial. To avoid the chance of a single experiment and to increase the reliability and persuasiveness of the experiment, the experiment was repeated 10 times at different speeds.

Figure 15a shows the error of ten experiments with different speeds of axial delivery of the catheter. The average error of catheter delivery is 0.95 mm, and the maximum error is less than 2.0 mm. The axial displacement of the catheter in this design is driven by the stepper motor to drive the catheter, which clamps the catheter’s forward and backward delivery. The possible causes of the error of the catheter axial delivery include the inertia of the slide table, loose and tight assembly between parts, and motor miss-steps.

Figure 15b shows the error of the guidewire delivery experiment at the different speeds. The average error of axial guidewire delivery is 1 mm, and the maximum error is less than 2.0 mm. The axial displacement of the guidewire is achieved by the stepping motor with a friction wheel rolling to deliver the wire back and forth. The error of the axial delivery may be caused by a machining error of the mechanical structure and the error caused by the fit of parts. In conventional interventional procedures, the surgeon’s manipulation error in radial delivery is close to 2 mm [28], so we believe that the delivery error of the robotic system is acceptable.

**Figure 16:**
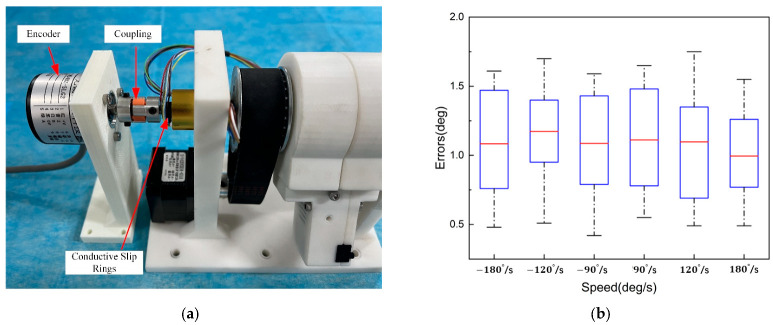
Catheter rotation unit verification experiment. (**a**) Experimental platform construction. (**b**) Experimental error.

As shown in Figure 16a, the radial displacement accuracy evaluation experiment uses an optical encoder to measure the rotational angle information of the catheter manipulator as the catheter is manipulated radially. Since the clamp clamps the catheter without sliding when rotating, the radial experiment examines the radial performance by measuring the displacement of the optical encoder axis driven by the rotating unit. A 5 mm shaft is passed into the inner shaft of the conductive slip ring of the conduit manipulator and secured. The other end of the shaft is connected to the metal shaft of the optical encoder by a coupling.

In the experiments, we set the speeds of 90°/s, 120°/s, and 180°/s to drive the robot to rotate clockwise/counterclockwise for one week and measured the motion error of the robot separately. To avoid occasional errors, the experiments were repeated ten times for each speed. Figure 16b shows the range of the error; the maximum value of the error is less than 2°, and the average error of the option is 1.09°. The rotation motion is just to adjust the direction of the tips of the catheter, and the risk of damaging the vascular is small during the operation [29].

### 4.3. Master–Slave Control Experiment

**Figure 17:**
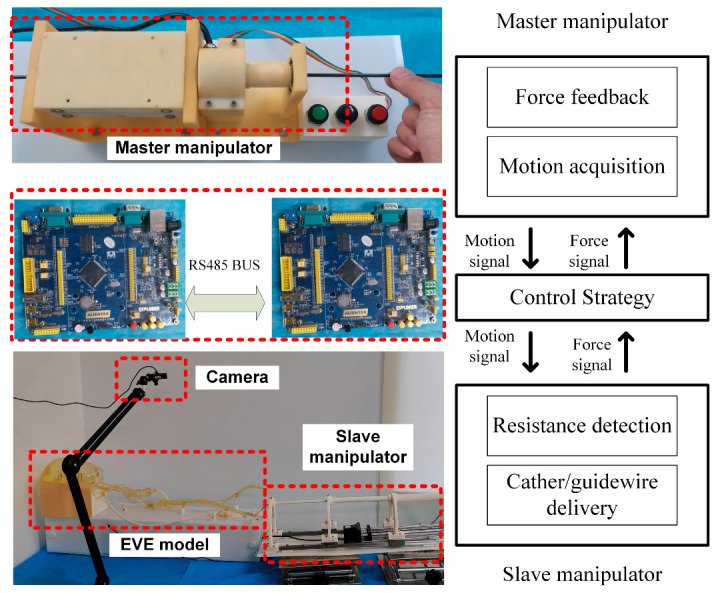
Experimental environment setup.

**Figure 18:**
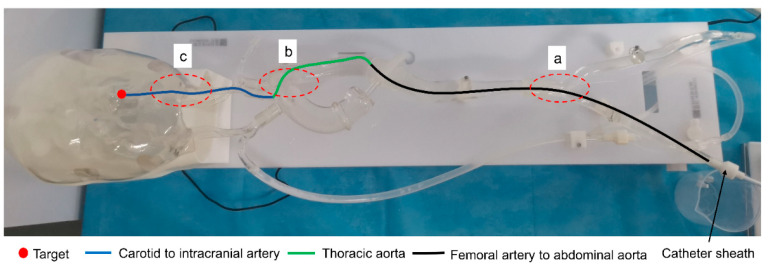
The in vitro model and experimental pathway. (**a**) Femoral artery to abdominal aorta junction. (**b**) Thoracic artery to carotid artery junction. (**c**) Carotid artery to target junction.

To verify the effectiveness of the proposed robotic system, we conducted experiments using a 1:1 in vitro model. The experimental scene is built as in Figure 17. The in vitro model is equipped with a circulating integrated pump, which can simulate blood flow. Figure 18 illustrates the in vitro model and experimental pathway. In the experiment, the surgeon manipulates the master robot in the isolation bin and controls the slave robot via teleoperation to complete the catheter/guidewire delivery. To simulate the surgical environment, a high-definition camera is set up at the model and provides visual feedback to the surgeon through the display to simulate medical fluoroscopy. Two microcontrollers are used to provide visual feedback and process data.

**Figure 19:**
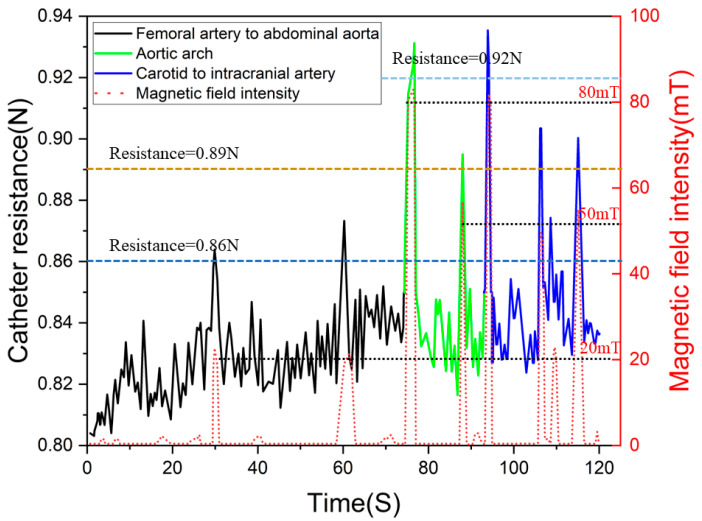
The in vitro model control experimental data.

Figure 19 shows the data of the experimental procedure, when the catheter moved to branch a (as in Figure 18). In the experiment, we set three levels of force feedback strategies. When the force sensor at the slave manipulator detected a catheter resistance of more than 0.86 N, we passed a current into the master device to create a resistance of approximately 1.8 N to alert the surgeon that the catheter was being resisted, but that the procedure could continue. When the slave force sensor detected resistance above 0.89 N, the master device was fed with a current to create a resistance of approximately 2.5 N to alert the surgeon that the catheter was currently under greater resistance. When the force sensor at the slave detected resistance above 0.92 N, the master device passed a current that generated a resistance of 3.5 N, at which point, the surgeon felt an increase in push-in resistance and would consider adjusting the catheter posture and reinserting it.

## 5. Discussion

In this paper, we developed a haptic feedback device for vascular interventions robots. Compared to traditional robot surgery devices, our device does not change the operating habits of the conventional clinical surgeon, allowing the surgeon to adapt more quickly to the robot’s operation. To reproduce the force on the catheter during surgery, we use MRF with a disc to achieve the reproduction of resistance. We analyzed the movements of the traditional surgeon operating the catheter and achieved the axial/radial movement acquisition of the catheter to maximize the retention of the surgeon’s clinical skills.

To verify the guiding effect on the magnetic field of our developed device, we used finite element analysis software to analyze the magnetic field. The magnetic field orientation analysis shows that the device can direct the magnetic field at an oblique angle through the MRF. The analysis shows that the magnetic field distribution of the developed equipment meets the design requirements. In addition, a test experiment was used to verify the performance of the device, which showed that the device provided a feedback force of approximately 1.5 N–4.65 N, which could provide a valid reference for surgeons.

We conducted experiments to determine device resistance, selected delivery speeds commonly used by surgeons, and measured the feedback resistance at different currents. We found that when the delivery speed is low, the change in speed has a slight effect on the drag. Compared to previous devices, the developed device has greater and clearer feedback force. Then, we completed the in vitro model experiment using master–slave control, and the experimental data showed that when the slave catheter rapidly exceeded the set threshold, the master could rapidly pass a current to the coil to generate a feedback force, verifying the effectiveness of the device. Compared to the feedback alerts generated by vibration, the device we developed is more intuitive in terms of feedback power and can increase a surgeon’s sense of immersion.

## 6. Conclusions

This work addressed the need for haptic feedback in interventional vascular surgery field. To characterize the force conditions of the intravascular catheter, we used magnetorheological fluid smart materials to develop a feedback device that not only provides haptic feedback but also captures the surgeon’s manipulation of the catheter. We use a disc immersed in MRF to generate resistance, which simplifies the structure of the device, reduces the size of the device, and facilitates the commercialization of the device. Some experiments have been of value to the developed equipment. Experiments have shown that our devices have very clear haptic feedback, which can help surgeons make decisions and improve their clinical skills.

## Data Availability

The data is unavailable due to privacy or ethical restrictions.
